# Inflammation of the Dental Pulp

**Published:** 1871-04

**Authors:** Jas. H. Ludwig


### AMERICAN JOURNAL

OF

### DENTAL SCIENCE.

Vol. IV. THIRD SERIES--APRIL, 1871. No. 12.

### ARTICLE I.

### *Inflammation of the Dental Pulp.*

[From the German of Prof. Dr. C. Wedl.]*

By JAS. H. LUDWIG, M.D., D.D.S.

When we bear in mind the many accidents to which the teeth are liable, we are really surprised that primary or idiopathic inflammation of the dental pulp does not occur more frequently than is actually the case. J. Tomes thinks, that even in as many as 99 cases in the 100, disease of the pulp as a result of caries is dependent on perforation of the pulp-cavity. Heider, also (Zeitschrift der k. k. Gesellsch. der Wiener Aerzte 1846 : abhandl. über Zahnschmerz) has written in a similar manner.

The existence of an idiopathic inflammation is determined with difficulty, and this difficulty is increased when the diagnosis has to be made by exclusion often difficult to do on account of established caries, and also when a developing periostitis may be taken for a pulp inflammation. A third difficulty is found in the difference between a commencing inflammation, and that state of congestion produced by hyperæsthesia of the pulp. In the absence of objective

* Pathologie der Zahne, mit besonderer Rucksicht auf Anatomie und Physiologie.

appearances, we are obliged to depend upon the subjective altogether, while its termination can only be known by its course. If decided intermissions of the pain occur, and if hyperæsthesia be present in other organs, we will have good cause for excluding inflammation.

If we observe carefully inflammation which is the result of so-called perforating caries, and take into consideration the preceding pain, we find that it is at first local, but afterwards invades the neighboring teeth, and even radiates over the entire side of the face.

The partially exposed pulp is acted upon by outside influences ; cold air, cold water, and acid substances act upon it with pain, while pressure upon the affected tooth produces a sense of relief, provided the exposed part of the pulp has not been pressed upon. The intensity of the pain varies according to the constitution and age of the individual, and frequently increases during pregnancy and the menstrual periods. In most cases the inflammation becomes suppurative, which we can see at the upper surface of a part of the pulp, and often at last we have an oedematous appearance, or it is covered with a lymphy, sticky flow. If there is no hindrance opposed to the discharge of the formed pus, a marked remission of the spanning, drawing or throbbing pain occurs, and even a decided intermission may take place, so that the patient will only be reminded of his sick pulp by some particular circumstance. The acute form then passes into the chronic.

If the pulp cavity has not yet been opened by the carious process, and only a thin portion of dentine remains as a protective covering to the pulp, it is readily influenced by outward agents of medium severity, and we have by this condition of things what dentists term, *odontalgia nervosa ex carie.* Inflammation, says Heider, does not seem to be present, for the paroxysms of pain occur frequently with great severity without any foreboding, but are often separated by entirely painless intervals. Cold impressions which in other cases of inflammation diminish the pain, increase it in these. Gradually warm and cold impressions cause a slight, temporary pain, on which account the patient is careful to avoid hot and cold food and drink, while that side of the jaw upon which the carious tooth is located becomes useless for mastication, on account of the intense pain produced in the act of biting, by food being forced into the decayed cavity. After this state of things has existed for a longer or shorter time, there generally follows after the pulp-cavity has been broken into, instantly such a severe pain, that tears flow from the eyes, and the patient feels as if it were impossible to endure the pain, did it last for any length of time. In a few minutes the pain lessens and becomes more bearable, but is still very threatening, and may continue for hours with ex ascerbations and remissions. It finally ceases gradually, and we generally have a short, painless interval, but the pain soon recurs, although no outside influences may be acting upon the nerves of the exposed pulps. The patient, however, does not always remain free from intense pain until exposure of the pulp occurs ; frequently, after a few tempory pains of moderate severity have once occurred, there may be produced from very slight causes such an intense and continuous tooth-ache as to last for hours together.

On pulps which have been exposed by penetrating caries we distinguish a partial as well as total iuflammation. The first is to be sought for particularly in such cases where the performation of the hard substance is limited to a small extent, and attacks only a part of the body of the pulp, perhaps the point of a papilla of a bicuspid or molar tooth.

The most prominent symptom is redness, proceeding from either over-fullness of the blood vessels, or extravasation into the pulp-tissue of blood-coloring matter, differing from that of the blood corpuscles. Between the congestive and diffused redness stands the hæmorrhagic, which appears red-sprinkled, formed in p ints or spotted. The boundaries of ihe extravasation gradually lose themselves in the parenchyma of the pulp. In partial inflammation the redness extends only to a section of the pulp-body, diminishing from the periphery toward the centre; it embraces either the one or the other of the root-pulps or only their broad parts. The intensity of the redness fluctuates between a weak rose-red and a deep blood-red, often with a mixture of gray which more or less preponderates. From ædematous swelling the inflamed pulp presents a yellow-red appearance; and from superficial suppuration a pale, greenish yellow color.

The increase in volume of an inflamed pulp is effectively prevented by the hard, resisting substance in which it is found, and it is a matter of general opinion, that one of the causes of the severe beating and lancinating pains, is the resistence of the dentine. When we are certain that inflammatory tooth-ache suffers modification through local circumstances, we believe, although otherwise reminded, it to be intensive neuralgia under the most different anatomical relations. Swelling of the pulp in partial inflammation is limited to the one or the other surface, but in many instances it is well-nigh impossible to locate a partial inflammation. At best we only succeed in such cases where the inflammation occurs in an atrophied pulp.

In regard to duration, we distinguish an acute form a chronic inflammation, as is well known, by its different degrees of intensity. The more acute the course of the inflammation the more rapid will be the destruction of the panenchyma; the more chronic its course the more gentle will be their intervals; in the first case the consistency of the organ is perceptibly diminished, and may even run into a soft, pulpy state.

The suppurative process is nearly always developed as a sequel of caries, and is limited to the upper surface-layer or to the root part of the pulp which is in relation to the carious region. The suppuration appears as a greenish-gray or greenish-yellow, semi-fluid, stringy covering, which contains pus-corpuscles as its characteristic morphological ingredient. The contents of these are in most cases affected with fatty degeneration, so much so that on being treated with acid, their granules do not appear so distinctly, on account of the fatty granules, and not unfrequently are the corpuscles so shrivelled and disintegrated that it is well-nigh impossible to distinguish them as such. The fluid inorganic matter of the suppurating mass is clouded with suspended fat-drops, which also appear aggregated in heaps of fat-granules. Under the influence of acid the mucuous is precipitated and be comes in form an out stretched, thready, persistent mass.

We often meet with cases where, in the superficial and apparently suppurrative substance of the pulp not a single pus-corpuscle is found, but a mere agglomeration of shrivelled, obscure granules, which appear imbedded in a precipitated mass, resembling the white of an egg.

If we examine into the deeper layers of that part of the pulp infiltrated with pus, we are easily convinced that a process of rapid growth of the parencyma-cells in acute progressive cases, can be traced far into its interior and even deep into the pulp-roots Sections of suitable pulps that have been imbedded in paraffin, lime or other similar substance, answer for this purpose. It is the tissue-cells of pulps, particularly in young persons which are subject to a high grade of prolification. We often meet these with double-granular cells partly round and partly spindle-formed, in company with other manifold gliding and variously formed cells, so plentifully represented that they crowd upon each other, and the more as we approach the inflamed mass.

Finally we only see the element-organs still more pressed on each other, and covered over, which assume mostly a round granular appearance, and exist enclosed in a layer of protoplasm, which gradually becomes smaller.

If we observe the blood vessels, we can diagnose texture-changes in the inflamed masses at the point of over-flow, as follows: a swelling and loosening of the connective-tissue sheaths of the small arterial and venous vessels and also a cloudiness of the sheaths, proceeding from scattered or agglomerated fat-granules, or from numerous, sometimes large, sometimes small, often grouped granules, which frequently cover the whole vessel entirely around, so much so that its characteristic appearances are lost, and can only be recognized as such by its continuation. Albrecht (Krankheiten der Zahnpulpe, 1858,) has called our attention to the considerable increase of volume, and the crooked and the greatly wounded course of the vessels.

We observe in inflammations which have become chronic, particularly, that the increased, long-continued, and oft-repeated blood-pressure upon the pulp, destroys the elasticity and centractility of the walls of the vessels; the element-organs of the connective tissue proliferate as soon as the vessel loses a certain mass of its usual succulent support. After expansion has occurred it can regain neither its first measure in length nor width; it becomes longer and wider, a bending-off sideways and often a varicose extension occurs, so that when it lies on the upper surface, bursts and bleeds easily, and occasions in the deeper part, the above mentioned extravasation. This state of things gives rise in its course to pigmentary and clouded appearances. If the vessels continue extended it is possible for the circulation to become interrupted, the changes of matter in their usual masses cannot go on, the matter which ought to be thrown off remains behind, which still further deranges its function.

The red blood-corpuscles as well as the white become necrosed by the stagnation of the column of blood; the first give over their coloring matter to the fluid around them, the pulp becoming thronged with blood coloring matter.

Texture changes also show themselves in the nerve-tube bundles near the inflamed masses. The most prominent symptom is their cloudiness, conditional upon their containing fat-corpuscles; which are sometimes scattered, sometimes aggregated and lying between the nerve tubes. (Neutritis.) The metamorphosis of the fatty-granules of the nerve-marrow is not proportionately spread out in a nerve bundle, so that the marrow of single nerve tubes may be free from fatty degeneration. In the connective tissue sheaths of the nerves we meet with the analogue of the vessels; that is groups of oval or round granules, pointing out a prolification of the element organs of the connective tissue.

We infer from this that the antecedents of inflammation in the nerve bundles of the pulp, whether followed by fatty degeneration of the nerve-marrow, or cell-increase of the instertitial connective tissue of the bundle, may have as a sequel through some painful impression, the destruction of the conducting medium in the axis cylinder.

The dentine cells in the vicinity of the inflamed mass suffer a fatty granular cloudiness of their protoplasm; their cohesion becomes weakened, and they gradually approach a total disintegration.

In acute spreading cases the fatty change occurs more intensively and extensively than in the chronic, and conjointly with this change proceeds the loosening of the pulp-tissue, which extends until reduced to a fluid state, so that there remains only a small part of discolored skin adhering to the pulp wall; extending also into the root-canal, and causing the rest of the pulp to be shriveled.

If we examine the entire aspect of an inflamed pulp we thereby discover the anomaly of matter-change; through an increased tendency of blood-flow to the pulp and a diminished one from it, we have an increased transudation, a piling up of excretory matter; further we have through a prolification of cells, resembling the tissue support of the pulp and the connective tissue of the vessels and nerves, and also a production of masses of pus-corpuscles; or larger, but immediately shrivelled granules, which prolifications together bring about a partial or total destruction of the pulp-parenchyma.

Acute inflammation of the pulp sometimes runs into a gangrenous destruction, which generally follows caries of both the deciduous and permanent teeth, as well as after dental periostitis, but seldom occurs independently. Like in other organs we distinguish two different and distinct forms of gangrene—a so-called moist and dry gangrene.

The signs of the moist consist in a greasy deep-red, reddish-brown, reddish-gray, or greenish-gray color, a change of its consistence to a thick pulpy state, with a remarkably persistent and specifically gangrenous smell. The pulp occasionally passes into a broken-down, curdy, crumbling, disgustingly stinking mass.

The tissue changes are known as inflammatory when the gangrene has not proceeded too far. It will be instructive to begin our investigation in the pulp-root and proceed toward the pulp-crown. The divided nerves as also the divided vessels indicate the prolifications just described, which predominate in masses as we advance toward the gangrenous portion, and finally cover the entire field. Thus the fatty degeneration grows in rapidly increasing progression. The contents of the nerve tubes crumble in one place and another. The walls of the vessels take on a fine granular appearance, and disintegrate before they are macerated; the oblong granules of their layers of muscular fiber lie scattered through each other. At the point where total gangrenous destruction has taken place, we notice a greasy yellow, yellowish brown-molecular detritus, generally containing imbedded in it numerous long, bow-form-crooked, acid fat crystals, like those in the sputa from pulmonary gangrene.

By the so-called dry gangrene of the pulp, there remains behind only a dark-brown, red-brown, tinder-like, or cuticular, slightly moistened mass, which no more fills up the pulp-cavity—a gaseous-flow occupies the remainder of the cavity. As histological ingredients, the following are named: red-brown or dark brown cylindrical masses (necrosed blood) enclosed in yet recognizable tubes (blood vessels.) These masses are an agglomeration of greasy yellow or brownish granules, and small granules of pigment crystals of haemotoidin.

If caries has preceded in a pulp which has become gangrenous, perforation of the pulp cavity in most cases has not yet taken place. In particular cases sealing up of the cavity gives us the cause. Gangrene produced primarily, seems to affect particularly teeth affected by mechanical abrasion. Besides these causative agents it is not universally known that deleterious outward influences favor gangrene in an inflamed pulp.

When the red coloring matter of the blood corpuscles transudes through the undamaged walls of the vessels, or after the bursting of these walls it is drawn to the corpuscles, it penetrates the dentine and gives to the teeth a reddish color. (M. Heider und C. Wedl über geröthete Zahne; Deutsche Viertl. f. Zahnheilk, 1863.) This color generally appears at the neck of the tooth where the enamel and dentine meet. The redness in many cases extends only a short distance from the neck in the direction of the root, diminishes in color as it proceeds, and becomes at last a weak rose-red. In the other cases the redness is more intense and spreads itself towards the apex of the root, generally stronger on one side of the root than the other. The deepest color, however, can appear on a *part* of the root.

If we endeavor to ascertain from cut sections of the different layers of dentine the extent of the redness, we sometimes meet with a very weak color or none at all, upon the zone of dentine which lies against the pulp-cavity or the canal of the tooth, but there follows, however, a sometimes broad, sometimes narrow, briskly tinged middle zone, which undergoes a weaker coloring outwardly. The inner zone of dentine is tinged most lively in the middle.

Finely polished sections of the different zones of dentine give us the desired information in regard to the coloring substance which they contain. Even the dentine canaliculi from their sources to their finest ramifications are filled with red coloring matter, while the inorganic substance does not appear colored at all. The coloring matter here shows that *redness* which is common to the blood, and looks like a homogenious, ungranular, filled in substance of the canaliculi. That we may not be deceived in this regard, we have only to remember that such coloring on polished sections does not take place in normal teeth. It is well known that tinges resembling the above can be produced artificially, by treating dentine with carmine, alizarin, Berlinblue, blood-red, &c. It can also be easily decided by experiment that the cementum does not take up coloring matter. If we cause the pigment to penetrate the dentine outwardly from the pulp cavity the dentine becomes colored, while this is not the case when the coloring matter is confined to the root of the tooth—the cementum does not become tinged.

We have immersed dried human teeth, after their crowns were broken away, in fresh rabbit's blood with the addition of about a quarter part of water. To prevent the evaporation of the water a layer of oil was poured upon it. After the lapse of about forty-eight hours, the uncovered plains of dentine were more or less extensively colored with a rose red, sometimes weak and sometimes more intensive. The prepared sections showed in the canaliculi of the dentine, the red blood coloring matter. To solve the question whether the red color of the dentine proceeds from the interior of the canal of the root, or from the layer of cementum outwardly or inwardly, the following experiments are given:

A six inch glass tube of suitable width was inserted into the open pulp-cavity of a lower molar tooth, and filled with rabbit's blood. Further, entirely sound teeth, the apical formina of which were securely closed with sealing wax, were laid in fresh rabbit's blood. The results of both experiments were decisive. In the first, it was colored an intense red, while in the last experiment the teeth manifested no traces of color though numerous sections were made.

The circumstance that different layers of dention appear sometimes more and sometimes less reddened can be accounted for in the different conditions which cause it. It is to be observed that the middle zone of dentine is more numerously ramified with canaliculi than the inner ones.

The red color of the dentine can only be brought to our eyes by the addition of reddened conaliculi, which can be seen shining through the inorganic matter. Should different modifications occur in their transparency, the red appearance will be correspondingly modified. The occasional yellow-red, rose-red, gray-red or gray-reddish color of different teeth, and the shades of color in the different zones of dentine confined within certain limits, do not come perhaps from changes of the blood coloring matter, or from its diminished imbitition, (we can readily convince ourselves of the proportionate color of the candiculi by inspection), but stands, with respect to the transparency of the inorganic matter, in relation with dentine. In caries of the tooth, and atrophy of the tooth-pulp, the dentine has taken on, as we know, towards the pulp-canal and pulp-cavity, a greasy whiteness, mixed with grayish yellow; and indeed at all such points where caries has not yet penetrated. The diminished transparency of the inorganic matter associated with the above in the inner zones of dentine, prevents the coloring matter of the canaliculi from shining through. The appearance of the redness depends upon the condition of the reddened canaliculi.

If the dentine is so formed that the canaliculi present themselves shorter in extension than we will find that the redness is smaller than when laid out in their long extension, where the coloring matter meets our eyes in greater amount.

In the canaliculi of the dentine, which are enclosed in the ramifications of the dentine-cell processes, it takes on more nearly an imbitition than a capillary attraction.

The appearance of reddened teeth is limited chiefly to such as are carious, whose pulps show a high red color or extravasation, and which impart their condition to the red colored place of the dentine. It is also known that when incisor teeth are treated with arsenic for the purpose of filling, their crowns become rose-red in the course of a few days, and may continue so for months after the filling of the tooth. This color often comes on without any previous signs, but often under appearances of congestion, always however brought about on teeth whose pulps were not removed by the nerve paste. It remains to say that red colored teeth have also been observed as death symptoms after cholera, typhus, and suffocation. Rose-red teeth with variations of their color at different times occur also as variation of formations. (Heider aus der Praxis; Deutsche viertlj. f. z., 1862.)

---

